# Supplementary material for: On the forbidden graphene’s ZO (out-of-plane optic) phononic band-analog vibrational modes in fullerenes
Source: Commun Chem. 2021 Jul 5;4:103. doi: 10.1038/s42004-021-00540-z (PMC9814469; doi:10.1038/s42004-021-00540-z)
Supplement: Supplementary file 2 — Supplementary Information [file 42004_2021_540_MOESM2_ESM.pdf]

Supplementary Information:
On the forbidden graphene's ZO (out-of-plane optic) phononic band-analog vibrational modes in fullerenes

Jesús N. Pedroza-Montero,[1] Ignacio L. Garzón,[2] and Huziel E. Sauceda[3,4,5,*]

[1] *Programa de Doctorado en Nanociencias y Nanotecnologías, CINVESTAV, Av. Instituto Politécnico Nacional 2508, México*

[2] *Instituto de Física, Universidad Nacional Autónoma de México, Apartado Postal 20-364, 01000 CDMX, México*

[3] *Fritz-Haber-Institut der Max-Planck-Gesellschaft, Faradayweg 4-6, 14195, Berlin, Germany*

[4] *BASLEARN, BASF-TU joint Lab, Technische Universität Berlin, 10587 Berlin, Germany*

[5] *Machine Learning Group, Technische Universität Berlin, 10587 Berlin, Germany*

SUPPLEMENTARY NOTES

1. Comparison between pristine and defective nanostructures

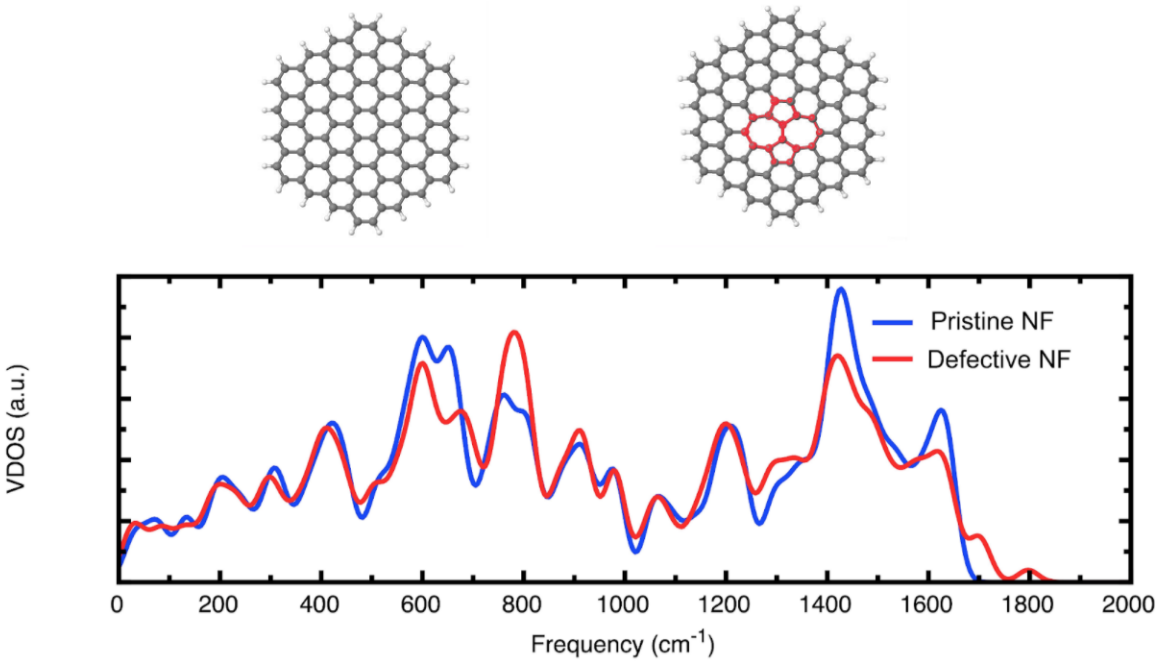

Supplementary Figure 1: Top panel: Left: Optimized structure of a graphene pristine nanoflake. Right: Optimized structure of a graphene defective nanoflake. In the latter case, two pentagonal-heptagonal defects were inserted at the center of a pristine nanoflake with 96 carbon atoms. Hydrogen atoms were used to saturate the surface dangling bonds. Bottom panel: VDOS of the pristine (blue curve) and defective (red curve) carbon nanoflakes.

* sauceda@tu-berlin.de

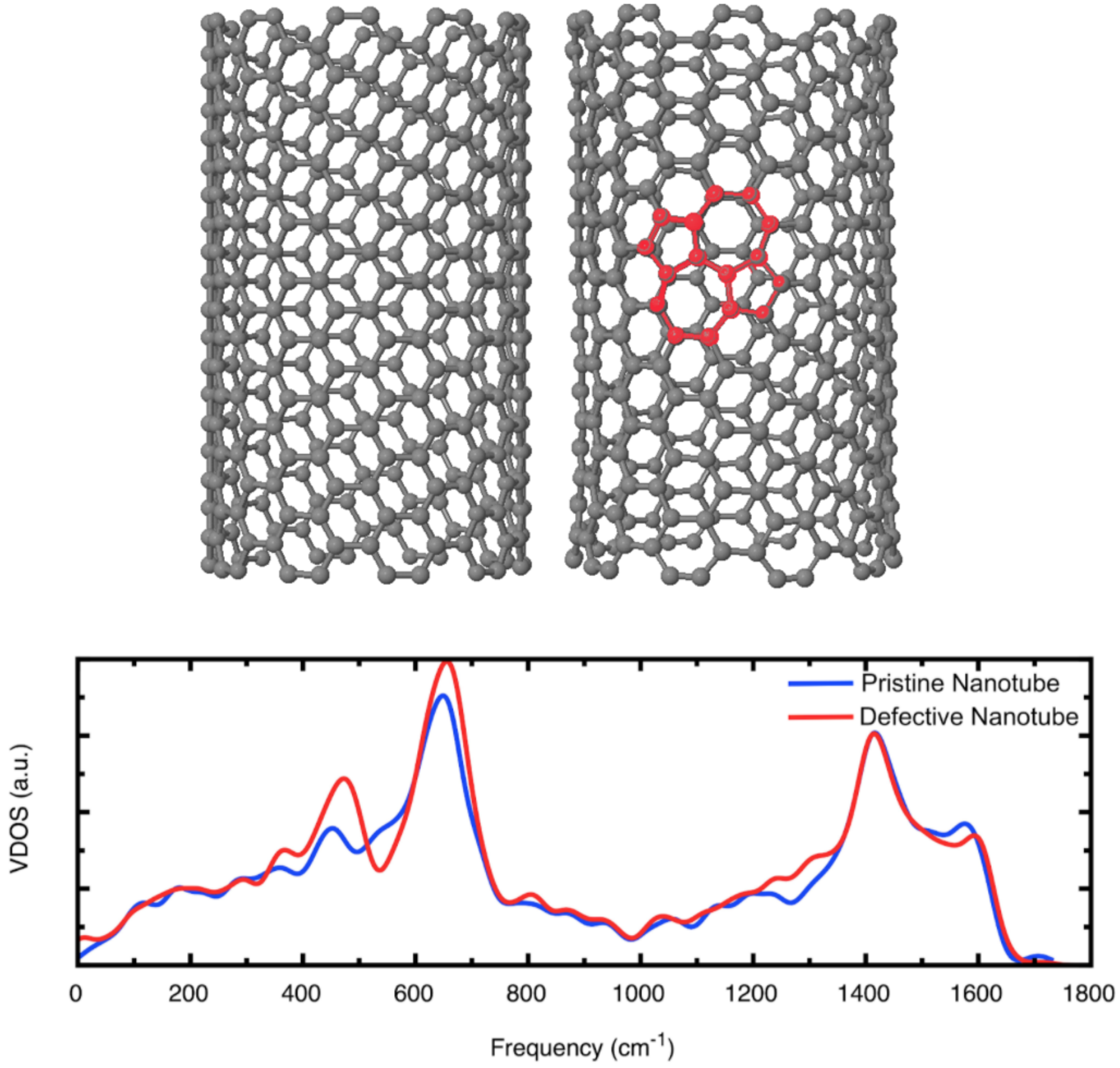

Supplementary Figure 2: Top panel: Left: Optimized structure of a pristine nanotube. Right: Optimized structure of a defective nanotube. In the latter case, two pentagonal-heptagonal defects were inserted at the surface of a pristine nanotube with 400 carbon atoms. Bottom panel: VDOS for the pristine and defective C400 nanotube.

2. Comparison of different shapes

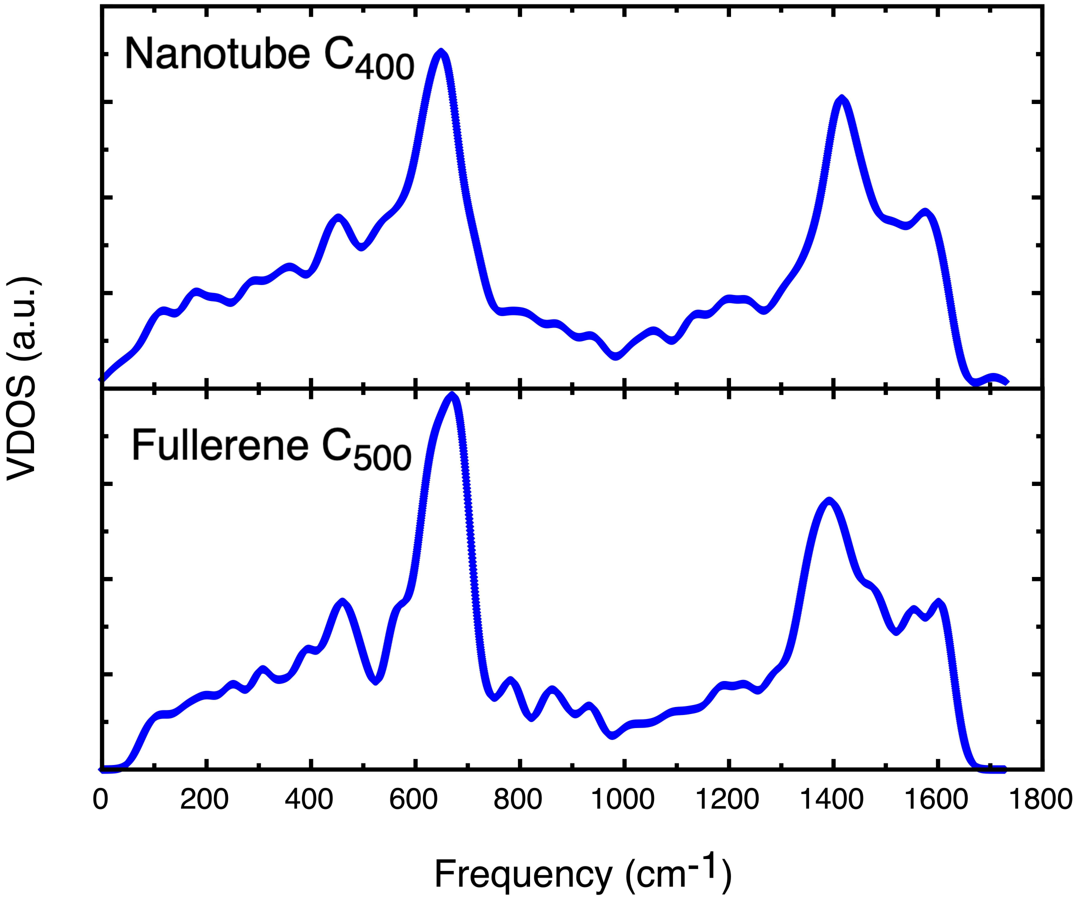

Supplementary Figure 3: Comparison between the calculated VDOS for the C500 fullerene and a pristine (without pentagonal defects) nanotube C400. This comparison indicates that the main differences in the VDOS profiles appear in the 400-1000 cm-1 region. In particular, the intense peak at 680 cm-1 for the fullerene C500 decreases in intensity for the C400 nanotube. It is concluded that the main reason for these differences in the VDOS might be attributed to the presence of pentagons in the fullerene, although the geometric structure also would induce other variations in the VDOS profiles. The vibrations in the C400 nanotube with frequencies around 700 cm-1 correspond to highly symmetric out-of-plane and twisting modes.

Supplementary Table 1: Correlation measurement of the size of the fullerene (number of atoms) with the evolution of the shape of the max freq. Z mode eigenvector: ⟨Atomic-amplitude-in-pentagon⟩/Max-Amplitude. Here, we have taken the mean of the atomic displacement amplitudes at the pentagonal faces and divided it by the maximum atomic oscillation amplitude in the eigenvector to compute their ratio. This shows that indeed the relative amplitude of the atomic oscillations around the pentagonal faces decrease as the fullerene size increases.

**#Atoms**	**Ampl-pentag-face/Max-Amp**	**Frequency (cm^{-1})**
60	0.515	710.63
180	0.207	696.46
240	0.373	678.44
260	0.102	684.62
500	0.069	699.71
720	0.096	713.04
